# Discovery of Acyl-Surugamide A2 from Marine *Streptomyces albidoflavus* RKJM-0023—A New Cyclic Nonribosomal Peptide Containing an N-ε-acetyl-L-lysine Residue

**DOI:** 10.3390/molecules29071482

**Published:** 2024-03-27

**Authors:** Zacharie A. Maw, Bradley Haltli, Jason J. Guo, Donna M. Baldisseri, Christopher Cartmell, Russell G. Kerr

**Affiliations:** 1 Department of Biomedical Sciences, Atlantic Veterinary College, University of Prince Edward Island, Charlottetown, PE C1A 4P3, Canada; zmaw@upei.ca (Z.A.M.);; 2 Nautilus Biosciences, Croda Canada Limited, Charlottetown, PE C1A 4P3, Canada; 3 Department of Chemistry & Chemical Biology, Barnett Institute for Chemical and Biological Analysis, Northeastern University, Boston, MA 02115, USA; 4 Bruker Biospin Corp., 15 Fortune Drive, Billerica, MA 01821, USA; 5 Department of Pharmacology, Comprehensive Center for Pain & Addiction, College of Medicine, University of Arizona, Tucson, AZ 85724, USA; 6 Department of Chemistry, University of Prince Edward Island, Charlottetown, PE C1A 4P3, Canada

**Keywords:** *Streptomyces*, surugamide, cyclic peptide, nonribosomal peptide, acyl-surugamide A2, cyclic nonribosomal peptide, marine *Streptomyces*, GNPS, natural products

## Abstract

We report the discovery of a novel cyclic nonribosomal peptide (NRP), acyl-surugamide A2, from a marine-derived *Streptomyces albidoflavus* RKJM-0023 (CP133227). The structure of acyl-surugamide A2 was elucidated using a combination of NMR spectroscopy, MS2 fragmentation analysis, and comparative analysis of the *sur* biosynthetic gene cluster. Acyl-surugamide A2 contains all eight core amino acids of surugamide A, with a modified N-ε-acetyl-L-lysine residue. Our study highlights the potential of marine *Streptomyces* strains to produce novel natural products with potential therapeutic applications. The structure of cyclic peptides can be solved using MS2 spectra and analysis of their biosynthetic gene clusters.

## 1. Introduction

Surugamides are a family of cyclic peptides that were first isolated from a marine-derived *S. albidoflavus* [1,2]. They are characterized by their eight amino acid cyclic peptide structure, which includes four D-amino acid residues. The surugamide family includes several core members, the surugamides A–E [1] and G–J [3], albucyclone A–F [3] and acyl-surugamide A [3]. Several other natural products can be included in the surugamide family when extending the family to other cyclic peptides that are cyclized by the same type of unique standalone β-lactamase superfamily cyclase enzyme [4,5]. These related natural products are the surugamide F–F2 [4], cyclosurugamide F [4], desotamides [6,7], wollamides [8], ulleungmycins [9], and noursamycins/curacomycins [10,11]. These cyclic peptides range in size between six and ten amino acids in length and share the characteristic presence of at least one tryptophan or phenylalanine and a C-terminal glycine or D-amino acid [12]. A biosynthetic gene cluster was proposed for the surugamides from *Streptomyces* sp. JAMM992 by using next-generation sequencing to sequence the genome, AntiSMASH [13] to identify the cluster, and gene knockouts to confirm associated genes [14]. The cluster has four non-ribosomal peptide synthetase (NRPS) genes, *surABCD*, two for the core surugamide A and two of which are responsible for the biosynthesis of the structurally unrelated surugamide F-F2 [14]. Surugamide F was discovered as a linear peptide; however, it was shown that, using recombinant technology, the same cyclization enzyme, surE, is capable of cyclizing surugamide F into cyclosurugamide F [4].

The discovery of new bioactive cyclic peptide natural products is highly desirable for their potential pharmacological applications. Cyclic peptides are highly desirable due to their conformational rigidity and increased metabolic stability, making them more resistant to enzymatic degradation compared to linear peptides [15]. Cyclic peptides possess the ability to be orally available, which is a significant advantage over linear peptides that are readily degraded in the gut [16,17]. Surugamides have been found to have promising biological activities, including antifungal [3], antimicrobial activity [18], and antiproliferative CDK inhibitors [19], as well as being a cathepsin B inhibitors [1]. Acyl-surugamide A is another derivative of surugamide A that was isolated from *Streptomyces albus* J1074 and has been shown to have good antifungal bioactivity (IC_50_ 3.5 µM) against *Saccharomyces cerevisiae* [3]. There have been mentions of additional members of the family in several previous publications; however, their structures have yet to be elucidated [3,20].

Ultra-high pressure liquid chromatography paired with high-resolution mass spectrometry (UHPLC–HRMS/MS) working in tandem to acquire both MS1 and MS2 data have significantly enhanced the utility of untargeted metabolomic analysis approaches for the identification of related molecules in complex mixtures of NPs. The UHPLC–HRMS/MS data can be processed by Global Natural Products Social Molecular Networking (GNPS) to cluster group the related NPs together based on MS2 fragment patterns [21]. GNPS has emerged as an indispensable tool for small molecule dereplication by comparing annotated natural product fragmentation patterns [21,22]. Utilizing an untargeted metabolomic approach using GNPS, we were able to determine that marine *S. albidoflavus* RKJM-0023 produces trace amounts of several unidentified natural products structurally related to surugamide A [1]. Using molecular networks to identify known molecules is a quick way to find new analogues and expand the chemical space of natural products produced by an organism [23]. Herein, we describe the isolation and structural elucidation of acyl-surugamide A2, a new cyclic surugamide containing a rare N-ε-acetyl-L-lysine residue, that to the best of our knowledge has only been identified in a couple examples [3,24]. Utilizing 2D NMR data (HMBC, TOCSY, and HSQC) coupled with UHPLC–HR-ESI-MS/MS for structural fragment annotation, and biosynthetic gene cluster annotation, we were able to elucidate the structure of acyl-surugamide A2.

## 2. Results and Discussion

### 2.1. Targeted Isolation of Acyl-Surugamide A2 from S. albidoflavus RKJM-0023 Isolated from a Marine Tunicate

**Figure 1:**
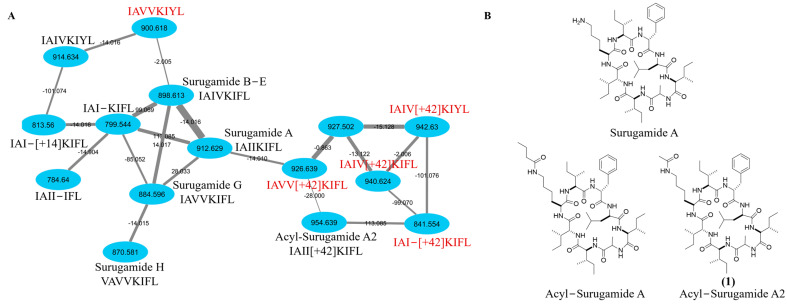
(**A**) Molecular network cluster generated by GNPS including the annotation of previously isolated surugamides A, B–E, G, and H. Nodes are labelled by their *m*/*z*, edges are labelled by the mass difference between neighbors, and edge thickness represents the cosine score between the MS1 ion (0.65-1). Each node is annotated with a previously predicted amino acid sequence (black) or our predicted amino acid sequence (red), where the + sign represents the addition of either [+14.02 Da], [+28.00 Da], or [+42.02 Da] to the following amino acid in the sequence. (**B**) Structure of surugamide A, acyl–surugamide A, and acyl–surugamide A2 (**1**).

Strain RKJM-0023 was isolated from the tunicate *Halocynthia papillosa* (Red Sea Squirt), collected from the Mediterranean Sea on the coast of Turkey. Analysis of the nearly complete 16S rRNA gene sequence (1456 bp) using the EzBioCloud 16S identification tool [25] determined that RKJM0023 was most closely related to *S. daghestanicus* NRRL B-5418^T^ and *S. albidoflavus* DSM 40455^T^ (99.86% identity). Chemical screening of fermentations of RKJM-0023 identified the production of surugamides and putative novel surugamide analogs in ethyl acetate extracts of fermentations conducted in BFM15m medium. The fermentation extracts were characterized by UHPLC–ESI-HRMS/MS and organized into molecular clusters using GNPS [21] resulting in the identification of a cluster with GNPS database matches to surugamide A and D (Figure 1). Additionally, two ions with *m*/*z* matching literature values for surugamide G and H [3] were also present within the cluster (Figure 1). The BFM15m fermentation extract was chosen for further investigation due to the production of significant quantities of the putatively novel surugamide analogue with an *m*/*z* 954.64.

The surugamide molecular network cluster from *S. albidoflavus* RKJM0023 was manually annotated based on the literature of both isolated and predicted amino acid sequences (Figure 1). Previously isolated surugamides A, B–E, G, and H were detected, while several other surugamide analogues have been previously predicted by cyclic peptide sequence algorithm NPMiner, including 914.63 (IAIVKIYL), 813.56 (IAI-[+14]KIFL), 799.54 (IAI-KIFL), and 784.64 (IAII-IFL) [26], while the *m*/*z* 926.64 (IAII[+14]KIFL), 940.62 (IAII[+28]KIFL), and 954.64 (IAII[+42]KIFL) were previously predicted using the MultiTag algorithm where they were referred to as reginamides [27]. Additionally, previously unreported surugamide associated ions (*m*/*z* 900.62, 927.50, 942.63, and 841.55) were included in the cluster. The *m*/*z* 841.55 has a distinct difference of 113.09, matching an isoleucine/leucine residue; therefore, the predicted amino acid sequence of IAI-[+42]KIFL lacking isoleucine is suggested. For *m*/*z* 940.62 and *m*/*z* 926.64, the MS2 fragmentation pattern compared to 954.64 suggests a similar +42-Lys residue with one or two Ile substitutions for a Val, similar to surugamide A valine substituted equivalents, surugamides B–E and G. The annotated sequences for *m*/*z* 926.64 and 940.62 contradicts the previously predicted amino acid sequences [27]; however, this is a different organism and therefore may be making different surugamide analogues.

The putatively novel surugamide *m*/*z* 954.64 analog was found to be most abundant in fermentations conducted in BFM15m, thus fermentations were scaled up in this medium (10 × 1 L). The fermentations were extracted with ethyl acetate and a combination of flash chromatography and reversed-phase HPLC were used to purify *m*/*z* 954.6385, named acyl-surugamide A2 (0.6 mg) (Figure S1).

### 2.2. Structural Characterization of Acyl-Surugamide A2 via NMR and UPLC-HR-ESI-MS/MS Fragment Annoatation

**Figure 2:**
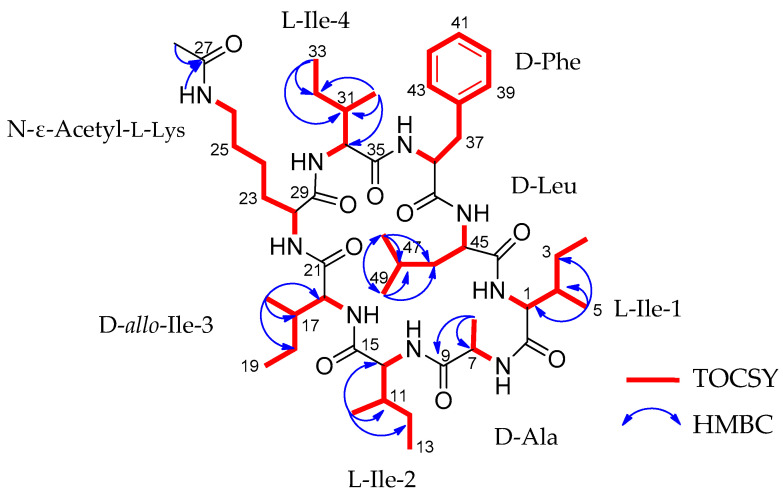
The TOCSY spin systems in red bold bonds and blue arrows indicate the measurable HMBC correlations for acyl-surugamide A2.

Acyl-surugamide A2 (**1**) (Figure 1, Table 1) was obtained as a white powder and HR-ESI-MS supported a molecular formula of C_50_H_83_N_9_O_9_, requiring 13 degrees of unsaturation. The peptidic nature of the compound was determined by analysis of the ^1^H NMR spectrum (Figure S2) which revealed the presence of nine amide protons, 50-NH (δH 7.13), 6-NH (δH 7.82), 9-NH (δH 8.29), 15-NH (δH 7.95), 21-NH-(δH 7.61), 26-NH (δH 7.75), 29-NH (δH 7.80), 35-NH (δH 8.44), 44-NH (δH 7.73), of which eight have a pairing alpha protons consistent with the molecular network identification surugamide A backbone, plus one amide bond. Analysis of 2D NMR HMBC, HSQC, and TOCSY (Figures S3–S5) spectra confirmed the identity of the amino acid constituents and revealed the presence of Ala, Leu, four Ile, Phe, and a unique acetyl-Lys residue (Table 1, Figure 2). To account for the remaining degree of unsaturation, it was determined that this compound must be cyclic. It was determined that this molecule cyclized in a peptide bond between the *N*-terminal and *C*-terminal amino acids as there are no hydroxyl groups, no shifts suggesting an ester-bond found in depsipeptide cyclization [28], and no aldehyde protons in the ^1^H. This is consistent with a surugamide A [1] core and a modification on the side chain residue of Lys, similar to what was observed with acyl-surugamide A [3]. The acetyl group methyl C-28 (δ_H_ 1.77, δ_C_ 23.1) is a distinct singlet with no TOSCY correlations. Due to the low amount of material, the HMBC spectra only presented correlations from each methyl group on the molecules, as well as the CH_3_ of the acetyl group to the carbonyl C27 (δ_C_ 169.4) and 26-NH (δ_H_ 7.75). The Lys secondary amide 26-NH shares a TOCSY spin system with H-26/H-25/H-24/H-23/H-22, placing it as the Lys residue functional group. This corroborates the MS/MS interpretation of the addition of a functional group extending from the Lys residue. Through careful review of the acquired NMR and comparison to the previously published NMR shifts for surugamide A [1] and acyl-surugamide A [3], all protons could be assigned to acyl-surugamide A2 (Table 1). To determine the sequence of amino acids, a combination of UHPLC–HR-ESI-MS/MS fragment annotation and biosynthetic gene cluster (BGC) analysis were used.

The amino acid sequence of cyclic peptides, such as the surugamides, can be determined using various methods. One strategy involves rebuilding the sequence based on the HRMS/MS predictable amino acid fragmentation of acyl-surugamide A2 amino acids and comparing it directly to the reference fragments of surugamide A in the GNPS library [21]. A similar reconstruction of the MS2 fragments was used to determine the sequence of surugamide A [1] and acyl-surugamide A [3] (Figure S6). Utilizing the information obtained from the NMR confirms that the molecule contains all eight core surugamide A amino acids with a modified acetyl-lysine (Table 1).

**Figure 3:**
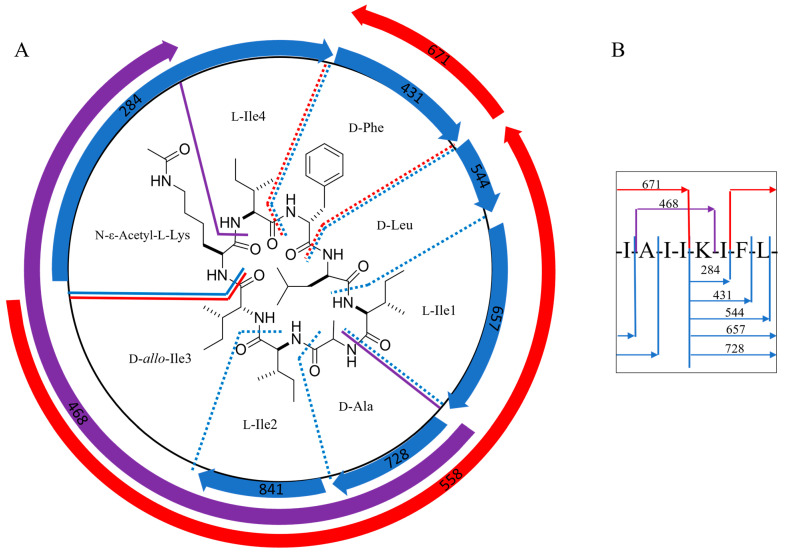
(**A**) The structure of (**1**) with diagnostic MS2 amino acid fragments of acyl-surugamide A2 was used to determine the amino acid sequence. (**B**) Amino acid sequence of acyl-surugamide A2 with fragments masses. The iterative addition of each amino acid on the fragment starting with acetyl-lysine (K) is in blue, the diagnostic fragment for AIIK is in purple, and the large matching surugamide A fragments confirming that K is the only modified amino acid is in red.

To determine the amino acid sequence of acyl-surugamide A2 using the MS2 fragments, the location of the three none-Leu/Ile amino acids must be determined; Ala (A, fragment mass difference of 71.04 *m*/*z*), Phe (F, fragment mass difference of 147.07 *m*/*z*), and the modified acetyl-Lys (aK, fragment mass difference of 170.11 *m*/*z*). The substitution of K for aK produces a mass difference of 42 *m*/*z*, accounting for the mass difference between surugamide A and acyl-surugamide A2. MS2 amino acid fragmentation of acyl-surugamide A2 was annotated by comparing the acquired fragments to the MS2 spectrum of surugamide A (Figure 3, Figures S6 and S7, Table 2). The annotated fragments match a full assembly of cyclo-[(I/L)A(I/L)(I/L)aK(I/L)F(I/L)] for acyl-surugamide A2. All fragments predicted to contain aK have complementary MS2 peaks in the surugamide A spectrum with a difference of −42 *m*/*z* (Figures S6 and S7, Table 2). For each additional *m*/*z* in the surugamide A cluster from *S. albidoflavus* RKJM0023 (Figure 3), the predicted amino acid sequence and composition were previously algorithmically predicted [26,27] or manually annotated based on their shared fragments to surugamide A and acyl-surugamide A2 (Figure S7).

### 2.3. S. albidoflavus RKJM-0023 Surugamide Biosynthetic Gene Cluster Analysis (sur)

The biosynthetic gene cluster of a NRP can also be used to determine the amino acid order of cyclic-NRPs [1,5]. To date, all core surugamides follow the LDLDLLDD amino acid configuration sequence [1,2,3,5,29] as well as the core AA order cyclo-[IAIIKIFL] with possible substitutions of L-Ile-1, L-Ile-2, or L-Ile-4 for Val [1,3,14]. The genome of *S. albidoflavus* RKJM-0023 was sequenced using PacBio RSII sequencing [30] and 1.5 × 10^6^ resulting reads were assembled into two contigs representing a 7,031,575 bp genome (GenBank accession CP133227) and a 90,910 bp plasmid (GenBank accession CP133227) [31]. The assembled contigs had a mean coverage depth of 1542X, an N50 of 7,031,575, and a GC content of 73.35%. The ends of both contigs were screened for regions of internal overlap; however, none were found, suggesting that both the genome and plasmid are linear. Annotation of the genome using the GenBank PGAP pipeline identified 6126 CDSs and 67 tRNAs.

**Figure 4:**
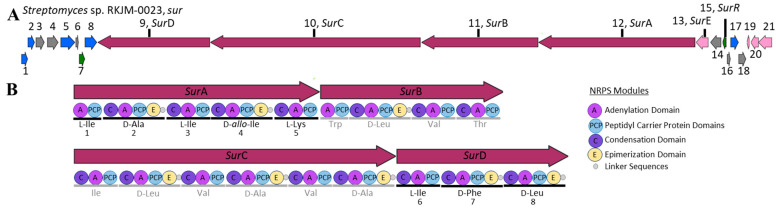
(**A**) Biosynthetic gene cluster organization of *sur* (surugamide cassette) from *S. albidoflavus* RKJM-0023, identified in contig 1 (region 4,039,078 to 4,121,529 bp). The coding proteins are sequentially numbered and color coded to indicate predicted function: core NRPS genes (red), regulatory (green), transport (blue), and additional biosynthetic genes (pink). Named *sur* genes are labeled; core NRPS *sur*A–D, penicillin-binding protein-type thioesterase *sur*E, and regulatory gene *sur*R. (**B**) The domain composition of each module in the core NRPS (*sur*A–D). The modules for the surugamide A core are in black and the surugamide F core in gray. Core NRPS module annotation from AntiSMASH 7.0.0.0 [33].

The draft genome sequence was mined for natural product biosynthetic gene clusters (BGCs) using AntiSMASH (7.0.0.0) [13]. Complete BGC identification was determined by a consensus rate of higher than 99% matching annotated BGCs in MIBiG [32]. Twenty-one BGC-containing areas were identified in the genome and none were detected on the plasmid. From the twenty-one BGC-containing regions, seven can be annotated due to high consensus to known BGC (≥99% identical) nucleotide sequences; these include SGR PTMs (BGC0001043), cyclofaulknamycin (BGC0002358), geosmin (BGC0001181), surugamide (BGC0001792), desferrioxamine B (BGC0000941), ectoine (BGC0000853), and antimycin (BGC0000958). One BGC was predicted to match the published surugamide cluster (*sur*) with a 100% consensus with the known cluster blast and MIBiG [32] reference sequence (Figure 4) (BGC0001792) [3,14,32,33].

The *S. albidoflavus* RKJM-0023 *sur* cluster contains twenty-one genes (Table 3) including the six *sur* genes has been previously established in the literature [3,14,34]. The core NRPS genes for the octapeptide surugamide A backbone are *surA* and *surD* [14]; combined, these two synthetases are the proposed core NRPS modules for the other surugamide analogues with modified Lys residues, acyl-surugamide A (butyryl functionalized Lys), and albucyclones A-F (albuquinone A functionalized Lys) [3]. The other two *sur* NRPS modules, *surB* and *surC*, are the synthetases for the biosynthesis of the separate decapeptide core of the surugamide F’s [14] (Figure 4). The additional biosynthetic gene *surE*, is the standalone cyclase domain with homology to a penicillin-binding protein-type thioesterase. *SurE* has been established to cyclase both the octapeptide and the decapeptide cores of the surugamides [5,34,35]. The Gnt-R transcriptional regulator, *surR*, has been shown to silence the *sur* gene cluster when expression is induced [3]. The function of the remaining 15 coding sequences of the *sur* BGC has not been formally established. The Pfam annotations for each *sur* protein coding region were annotated by BlastP [36] and are summarized in Table 3. The *sur* BGC from *S. albidoflavus* RKJM-0023 was compared directly to the *sur* BGC from *S. albidoflavus* J1074 (CP004370.1) and several other publicly available surugamide BGC sequences.

**Figure 5:**
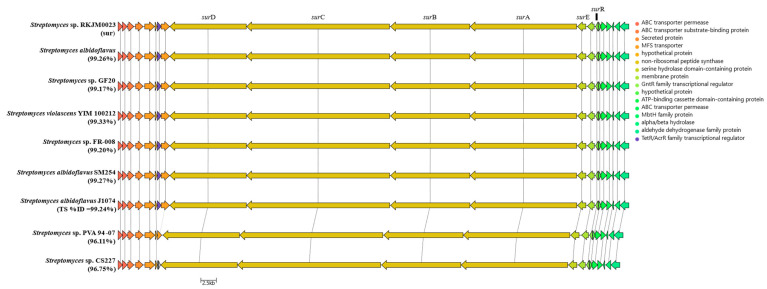
Comparative analysis of the *sur* biosynthetic gene cluster from *S. albidoflavus* RKJM-0023 (CP133227). Genes are color coded by proposed functions from the top hit on NCBI BlastP function. The nucleotide sequence identity of each *sur* is listed compared to RKJM-0023 and each cluster is predicted to synthesize surugamide demonstrating that the BGC is highly conserved.

The *sur* nucleotide sequence obtained from RKJM-0023 was directly compared to the *sur* BGC sequence from *S. albidoflavus* J1074 (BGC0001792 (MIBiG), NCBI accession CP004370.1). The nucleotide sequences had a high percent identity of 99.24% and a similarity score of 1.488 × 10^5^. The RKJM-0023 *sur* BGC sequence exhibits a high degree of gene synteny with previously published *sur* BGC sequence structures [4,14,37] with no additional modules and no significant deletions (Figure 5). The *sur* BGC identified from *S. albidoflavus* RKJM-0023 is a complete *sur* gene cassette, complete with the four core NRPS genes *surABCD*, the trans-acting PBP-type TE gene *surE*, and the regulator *surR* [4,5,14]. AntiSMASH detected epimerization (E) domains in modules 2 and 4 of *surA* and modules 7 and 8 of *surD* (Figure 4B). Furthermore, antiSMASH also predicted that condensation (C) domains from modules 3, 5, and 8 would accept D-configured substrates. The collinearity of E domains and D-accepting C domains is consistent with previously reported surugamide stereochemistry [3,14]. To verify that each epimerization domain was functional, the E domain amino acid sequences from modules 2, 4, 7, and 8 were extracted from the *sur* BGC of *S. albidoflavus* RKJM-0023 and compared to the homologous domains from *S. albidoflavus* J1071 and JAMM992 (Figure S8), as the stereochemistry of the surugamides produced by these strains were previously determined by Marfey’s analysis [1,3,38]. The *sur* E domains were aligned to the reference domains from modules 2 and 4 of the gramicidin BGC (BGC000367) and the conserved E domain active sited motifs (E1–E5) were annotated (Figure S8) [39,40,41]. The sequences of the E domain active site motifs were identical between the three strains for each module compared (Figure S8). This suggested that none of the *S. albidoflavus* RKJM-0023 *sur* E domains had acquired a mutation that would render any of the E domains inactive. While we did not determine the amino acid stereochemistry of acyl surugamide A2, the alignment of the pattern of the epimerization domains in *S. albidoflavus* RKJM0023 *sur* cluster suggests that acyl-surugamide A2 is consistent with the amino acid configuration pattern of L-D-L-D-L-L-D-D as previously described for all surugamide A cores [14]. The order of the modules corroborates the observed amino acid sequence determined by the fragmentation patterns by MS2 (Figure 3, Table 2). Acyl-surugamide A2 has a sequence of cyclo-[L-Ile-D-Ala-L-Ile-D-*allo*-L-Lys-L-Ile-D-Phe-D-Leu], established by MS2 and *sur* BGC analysis.

The biosynthetic origins of the acetyl-Lys in acyl-surugamide A2 is unknown. No acetylase was observed in the BGC (Table 3). Other potential routes of biosynthesis include using acetyl-Lys as a building block incorporated by a promiscuous *surA* adenylation domain in the fifth module of the NRPS from *surA*, or added post-NRPS biosynthesis by an acetylase after the surugamide core is made. Another potential root is nonenzymatic via acetylation by acetyl phosphate, a known method of widespread protein acetylation in *Streptomyces* spp. [42]. Analysis of the sur BGC genes and surrounding area revealed no identifiable acetyltransferase in the RKJM-0023 *sur* BGC (Figure 5); however, NCBI Prokaryotic Genome Annotation Pipeline identified 69 GNAT family N-acetyltransferase domains scattered throughout the genome [43]. Lysine acetylation in living cells, including *Streptomycetes*, is a ubiquitous and conserved post-translational modification in primary metabolism [44]; however, to the best of our knowledge there are no examples of a post-translational modification occurring on a natural product lysine residues. The acetyl-Lys post-translational modification is typically reserved for proteins and serves a critical and unique role in histone interactions as a central epigenetic control of gene transcription [45].

A comparative analysis of the *sur* BGC to nine other *sur* BGC protein sequences shows the highly conserved *sur* BGC in many *Streptomyces* spp. (Figure 5). The *sur* clusters with the highest percent identity were both marine isolates, *S. albidoflavus* YIM 100212 and SM254, having 99.33% and 99.27%, respectively. The *sur* BGC is highly conserved among *S. albidoflavus* isolates, with BiGFAM identifying 85 submitted sequences of the *sur* BGC and showing that all *sur* BGCs have been identified exclusively in *Streptomyces* species to date [46].

## 3. Methods and Materials

### 3.1. General Experimental for MS Analysis

A Thermo Scientific (Waltham, MA, USA) Vanquish UHPLC chromatograph equipped with HRMS-CAD-UV detection, which included a Thermo Scientific ID-X Tribrid mass spectrometer fitted with a heated electrospray ionization (H-ESI) source, a Thermo Scientific charged aerosol detector VF-D20-A, and a Thermo Scientific diode array detector (DAD) VF-D11-A-01 scanning 190–600 nm, was used. The solvents A = 0.1% FA in water and B = 0.1% FA in acetonitrile were used at a 0.5 mL/min flow rate with a Kinetex 1.7 μm C18 100 Å (50 × 2.1 mm) with the following gradient: 0 min = 5% B, 0.2 min = 5% B (isocratic), 4.8 min = 98% B, 8 min = 98% B (isocratic), 8.5 min = 5% B, 9.8 min = 5% B (isocratic). The MS parameters include positive ion scans performed from 150–2000 amu at an ion transfer tube temperature of 300 °C and a vaporizer temperature of 275 °C. NMR spectra were obtained on a Bruker (Billerica, MA, USA) AvanceNeo NMR spectrometer (^1^H: 600 MHz, ^13^C: 150.9 MHz) equipped with a 5 mm TCI cryoprobe. All chemical shifts (δ) are referenced to the DMSO-*d_6_* residual solvent peaks [^1^H (DMSO-*d*_6_): 2.50 ppm; ^13^C (DMSO-*d*_6_): 39.51 ppm]. Automated flash chromatography was performed on a Teledyne (Waterloo, ON, Canada) Combiflash Rf200 using C18 RediSep columns (24 g). HPLC purifications were carried out on a Waters Corporation (Milford, MA, USA) auto purification system coupled with an evaporative light-scattering detector and UV detector. All reagents were purchased from commercial sources and used without further purification unless otherwise stated.

### 3.2. Isolation of RKJM-0023

RKJM-0023 was isolated in April 2013 from a marine sample collected under a permit issued to Prof. Dr. Belma Konuklugil, Ankara University, Faculty of Pharmacy, Department of Pharmacognosy, 06100 Tandoğan Ankara (granted 02.01.2012 by the Ministry of Food, Agriculture, and Animal Husbandry, Directorate General on Agricultural Researches and Policies; Issue: B.12.0.TAG.0.04.03.730.10-2457). RKJM-0023 was isolated from the tissue of a tunicate *Halocynthia papillosa* collected in the Mediterranean Sea off the coast of Turkey (36.591415, 30.600488) at a depth of 18 m via SCUBA. The tunicate tissue was homogenized and serial dilutions were plated on raffinose histidine agar plates (raffinose 10 g/L, histidine 1 g/L, KH_2_PO_4_ 1 g/L, FeSO_4_·7H_2_O 0.01 g/L, noble agar 12 g, 1 L ddH_2_O with the pH = 7.5) supplement with Instant Ocean^®^ (Mystic, CT, USA) marine salts (18 g/L) [47], cycloheximide (50.0 µg/mL), and nalidixic acid (15.0 µg/mL) [48]. The plates were incubated at 22.5 ± 2.5 °C and strain RKJM-0023 was purified by serial subculturing. To identify the strain, the 16S rRNA gene was amplified and sequenced as described previously [49]. The 16S rRNA sequence (1456 bp) was analyzed using the EZBioCloud 16S rRNA classification tool (database ver. 2021.07.07) [25]. RKJM0023 was archived in a solution of 25% glycerol at −80 °C.

### 3.3. Fermentations and Extraction

A two-stage seed culture process was used to generate inoculum for fermentations. Approximately 50 µL of glycerol stock was used to inoculate 7 mL of BSM1m medium (dextrose 10 g/L, yeast extract 4 g/L, agar 0.4 g/L, soluble starch 15 g/L, calcium carbonate 1 g/L, NZ Amine A 4 g/L, Instant Ocean^®^ 18 g/L, pH 7.3) in a 25 × 150 mm culture tube containing five 4 mm glass beads and incubated at 30 °C and 200 RPM. After 24 h, 1 mL of the first-stage seed was transferred to 50 mL of fresh BSM1m broth and incubated under the same conditions for 24 h. For the small-scale media screen, 200 µL of the second stage seed was transferred to test-tubes containing 7 mL of one of the following media: BFM15m (sucrose 20 g/L, Bacto peptone 2 g/L, cane molasses 5 g/L, FeSO_4_·7H_2_O 0.1 g/L, MgSO_4_·7H_2_O 0.2 g/L, potassium iodide 0.5 g/L, calcium carbonate 5 g/L, Instant Ocean^®^ 18 g/L, in 1 L ddH_2_O) [50], BFM16m (glucose 40 g/L, dried yeast 5 g/L, K_2_HPO_4_ 1 g/L, NaCl 1 g/L, (NH_4_)SO_4_ 2 g/L, CaCO_3_ 2 g/L, FeSO_4_-7H_2_O 0.001 g/L, MnCl_2_-4H_2_O 0.001 g/L, ZnSO_4_-7H_2_O 0.001 g/L, NaI 0.0005 g/L, in 1 L ddH_2_O) [50], BFM17m (corn starch 10 g/L, pharmamedia 5 g/L, CaCO3 1 g/L, NaI 0.0005 g/L, in 1 L ddH_2_O) [50], BFM18m (glucose 40 g/L, casamino acids 15 g/L, NaCl 5 g/L, CaCO_3_ 2 g/L, K_2_HPO_4_ 1 g/L, MgSO_4_ 12.5 g/L, in 1 L ddH_2_O) [50], BFM19m (glycerol 30 g/L, corn syrup 15 g/L, pharmamedia 10 g/L, fish meal 10 g/L CaCO_3_ 6 g/L, in 1 L ddH_2_O) [50], BFM20m (molasses 60 g/L, soluble starch 20 g/L, fish meal 20 g/L, CuSO_4_-5H_2_O 0.1 g/L, NaI 0.0005 g/L, CaCO_3_ 2 g/L, in 1 L ddH_2_O) [50], BFM31m (modified PVA; maltose 20 g/L, Organotechnie Vegetal peptone ET1 10 g/L, V8 juice 100 mL/L, in 1 L ddH_2_O, and pH adjusted to 7.0 ± 0.2) [51], or ISP2m. Fermentations were extracted with 10 mL of EtOAc and concentrated for UHPLC–HRMS/MS analysis. For the large-scale fermentation, the second-stage seeds from multiple flasks were combined, and 10 mL of seed culture was used to inoculate each of 10 Fernbach flasks, each containing 1 L of BFM15m medium. After 5 days at 30 °C with shaking at 200 RPM, the cultures were extracted three times with equal volumes of EtOAc. The organic layers were combined and dried in vacuo.

### 3.4. Global Natural Product Social Networking (GNPS) Analysis of Family Members

The UHPLC–HR-ESI-MS/MS chromatograms obtained were converted from a .RAW file to an open-source MS file type .mzML using msConvert (ver. 3.0.18232), which is part of the ProteoWizard tool kit [52]. The .mzML file was then uploaded to the GNPS server using WinSCP (https://winscp.net/eng/download.php) (accessed on 1 April 2021). The classical molecular network was generated using Global Natural Products Social Molecular Networking (GNPS) [21]. Notable molecular network setting parameters included precursor ion mass tolerance of 2.0, fragment ion mass tolerance of 0.5, minimum pairs cos of 0.7, network TopK of 10, minimum matched peaks of 6, and minimum cluster size of 2. The molecular network was analyzed and visualized using Cytoscape (ver. 3.8.1) [52]. The surugamide molecular network cluster contained 11 unknowns, including GNPS fragmentation database matches to annotations of surugamide A and D [1], while also containing literature matching *m*/*z* values for surugamide G and H [3]. The analogues were then reanalyzed with Xcalibur, and their MS2 fragmentation patterns were compared to that of surugamide A, resulting in a total of 15 compounds within the Surugamide family produced by RKJM-0023. The acyl-surugamide A2 fragmentation pattern was manually annotated to determine the amino acid sequence.

### 3.5. Chromatographic Purification

The *S. albidoflavus* RKJM-0023 crude extract (350 mg) was prepared for solid load injection by adsorbing on C_18_ with initial fractionation performed using a 24 g C_18_ column (High-Performance GOLD RediSep Rf) using a mobile phase flow rate of 30 mL/min. The mobile phase consisted of a linear gradient from CH_3_OH:H_2_O (10%:90%) to 100% CH_3_OH over 30 min followed by 100% CH_3_OH for 5 min. Acyl-surugamide A2 was further purified via RP-HPLC using a Waters Corporation semi-preparative C_18_ column (SunFire C_18_ 100 Å, 3.5 µm, 4.6 mm × 150 mm). Isocratic elution with 48% H_2_O containing 0.1% formic acid and 52% CH_3_OH containing 0.1% formic acid was used over 40 min. The eluent was monitored by ELSD and MS at *m*/*z* 954.6. Acyl-surugamide A2 eluted as a single peak at 19 min. Subsequent evaporation in vacuo resulted in 0.6 mg of pure acyl-surugamide A2.

Acyl-surugamide A2 (1): white solid; UV (ACN) λ_max_ (log ε) 190, 210; ^1^H NMR (DMSO-d6, 600 MHz) and ^13^C NMR chemical shifts extrapolated from 2D HSQC data (DMSO-d6, 150.99 MHz) are described in Table 1; HRESIMS *m*/*z* 954.63851 [M + H]^+^ (calcd for C_50_H_83_N_9_O_9_, *m*/*z* 954.63865).

### 3.6. DNA Isolation, Genome Sequencing, and Biosynthetic Gene Cluster Analysis of RKJM-0023

Genomic DNA (gDNA) was isolated from *S. albidoflavus* RKJM-0023 using the DNeasy UltraCLean Microbial kit (Qiagen, Hilden, Germany) according to the manufacturer’s instructions. Biomass for DNA isolation was obtained by culturing RKJM-0023 in ISP2m medium for two days in ISP2m medium (yeast extract 4 g/L, malt extract 10 g/L, dextrose 4 g/L, supplemented with 18 g/L instant ocean, in 1 L of deionized water) at 30 °C and 200 RPM.

The gDNA was repurified with a DNeasy Power Clean Pro kit (Qiagen), followed by library preparation using the SMRTbell^®^ prep kit 3.0 protocol. The gDNA library was sequenced on a Pacific Biosciences Sequel II instrument using the adaptive loading protocol, Sequel II Sequencing Kit 2.0, SMRT Cell 8M and 30 h movies with a 2h pre-extension time by McGill University and the Genome Quebec Innovation Centre (Genome Quebec). The assembly was carried out using the HGAP4 workflow developed by PacBio (pb_hgap4 from SMRT Link v 11.0.0). The assembled genome was returned as two contigs; ctg.1 with 7,031,575 bp and ctg.2 with 90,910 bp. The genome was deposited in NCBI (genome CP133227 and plasmid CP133228) and annotated by the NCBI Prokaryotic Genome Annotation Pipeline (PGAP) [43]. The number GNAT family N-acetyltransferase domains were counted using the NCBI Genome Workbench (version 3.8.2) [53] to review the PGAP annotations. For localization of potential BGCs the consensus assembly sequence was annotated using AntiSMASH 7.0.0.0 [33], and the *S. albidoflavus* RKJM-0023 *sur* biosynthetic gene cluster sequence was extracted for further analysis.

Comparative analysis of the gene synteny and *sur* coding genes of the *S. albidoflavus* RKJM-0023 *sur* cluster was performed by gathering eight *sur* BGC sequences by searching for annotated surugamide clusters in NCBI [23] and selecting ClusterBlast matches from AntiSMASH [13]. Comparative analysis of the GenBank sequences of *sur* BGC was done using the clinker tool [54] and compared using BlastN for percent identity to the RKJM-0023 *sur* nucleotide sequence [36]. For epimerization domain analysis between *S. albidoflavus* RKJM0023 (CP133227) and previously stereochemical elucidated surugamide producing strains *S.* sp. JAMM992 (*sur*A AXN72677.1, *sur*D AXN72680.1) and *S. albidoflavus* J1074 (BGC0001792, CP004370.1), the protein sequences of each epimerization domain from the core surugamide A NRPS modules were aligned to reference epimerization domains from gramicidin BGC (BGC000367, AP008955.1) using MUSCLE [55] using Geneious Prime^®^ , https://www.geneious.com (accessed on 20 October 2023, ver. 2023.2.1).

## 4. Conclusions

Based on our findings, we have discovered a novel surugamide A analogue, acyl-surugamide A2, from a marine-derived *S. albidoflavus* RKJM-0023. The targeting of analogues is greatly simplified when using GNPS to group natural products based on their MS2 fragmentation patterns. Continued work will see acyl-surugamide A2 tested for biological activity. Our study highlights the potential of unique *Streptomyces* isolated from marine environments to produce novel natural products [56]. We also demonstrate that MS2 fragmentation patterns and analysis of biosynthetic gene clusters can be used to solve the structure of cyclic peptides, as previously shown in studies on surugamides [1,3,4]. It is also another demonstration of the utility of using GNPS-calculated molecular networks to highlight potential bioactive natural product analogues that can be streamlined for isolation [21,23]. Our study adds to the growing body of research on natural product biosynthesis and highlights the importance of exploring marine environments for the discovery of novel natural products with potential therapeutic applications. Future studies may choose to synthesize acyl-surugamide A2 using the established solid-phase peptide synthesis strategy [5,57] for further studies of the biosynthesis and bioactivity of acyl-surugamide A2 as natural abundance of the natural product is extremely low. Further, other surugamide analogues discoveries may lead to the development of new bio-actives.

## Figures and Tables

**Table 1:** NMR spectroscopic data (^1^H 600 MHz, ^13^C 150.9 MHz, DMSO-*d*_6_), TOCSY, and HMBC for acyl-surugamide A2 (**1**).

		Acyl-Surugamide A2 (1)			
Residue	Position	^a^ δ_C_ type	δ_H_ (*J* in Hz)	TOCSY	HMBC
L-Ile-1	50-NH		7.13, m	1, 2, 4, 5	
	1	57.7, CH	4.07, t (7.0)	50-NH, 2, 3, 4, 5	
	2	36.0, CH	1.77, m	50-NH, 1, 3, 4, 5	
	3	24.5, CH_2_	1.26, 1.11, m	1, 2, 4, 5	
	4	11.7, CH_3_	0.79, m	50-NH, 2, 3	
	5	15.6, CH_3_	0.79, m	50-NH, 2, 3	3, 2, 1
	6	ND, C			
D-Ala	6-NH		7.82, m	7, 8	
	7	48.45, CH	4.22, m	6-NH, 8	
	8	19.3, CH_3_	1.21, d (6.7)		9, 7
	9	^b^ 173.0, C			
L-Ile-2	9-NH		8.29, brd (7.44)	10, 11, 12, 13, 14	
	10	58.0, CH	4.16, m	9-NH, 11, 12, 13, 14	
	11	35.8, CH	1.75, m	9-NH, 10, 12, 13, 14	
	12	24.9, CH_2_	1.46, 1.12, m	9-NH, 10, 11, 13, 14	
	13	11.1, CH_3_	0.82, m	9-NH, 10, 11, 12, 14	
	14	14.9, CH_3_	0.82, m	9-NH, 10, 11, 12, 13	12, 11, 10
	15	ND, C			
D-*allo*-Ile-3	15-NH		7.95, m	16, 17, 18, 19, 20	
	16	56.9, CH	4.18, m	15-NH, 17, 18, 19, 20	
	17	36.7, CH	1.81, m	15-NH, 16, 18, 19, 20	
	18	26.2, CH_2_	1.30, 1.21, m	15-NH, 16, 17, 19, 20	
	19	11.9, CH_3_	0.81, m	15-NH, 16, 17, 18, 20	
	20	15.1, CH_3_	0.81, m	15-NH, 16, 17, 18, 19	18, 17, 16
	21	ND, C			
N-ε-Acetyl-L-Lys	21-NH		7.61, m	22, 23, 24	
	22	52.43, CH	4.27, m	21-NH, 23, 24, 25, 26	
	23	32.1, CH_2_	1.54, 1.41, m	21-NH, 22, 26, 26-NH	
	24	22.7, CH_2_	1.20, 1.13, m	21-NH, 22, 26, 26-NH	
	25	28.8, CH_2_	1.27, m	26, 22, 26-NH	
	26	38.93, CH_2_	3.01, 2.87, m	23, 24, 25, 26-NH	
	26-NH		7.75, m	22, 23, 24, 25, 26	27
	27	^b^ 169.4, C			
	28	23.1, CH_3_	1.77, s		27
	29	ND, C			
L-Ile-4	29-NH		7.81, m	30, 31, 32, 33, 34	
	30	58.3, CH	3.85, m	29-NH, 31, 32, 33, 34	
	31	36.2, CH_2_	1.43, m	29-NH, 30, 32, 33, 34	
	32	25.1, CH_2_	1.14, 0.81, m	29-NH, 30, 32, 33, 34	
	33	11.5, CH_3_	0.68, t (7.55)	29-NH, 30, 31, 32, 34	32, 31
	34	15.2, CH_3_	0.44, d (6.75)	29-NH, 30, 31, 32, 33	32, 31, 30
	35	ND, C			
D-Phe	35-NH		8.44, d (8.24)	36, 37	
	36	55.0, CH	4.38, m	35-NH, 37	
	37	36.8, CH_2_	2.68, t (12.57), 3.24, m	35-NH	
	38	^b^ 138.5, C			
	39, 43	128.6, CH	7.24, m	37	
	40, 42	129.6, CH	7.22, m	37	
	41	126.7, CH	7.17, m		
	44	ND, C			
D-Leu	44-NH		7.73, m	45, 46, 47, 48, 49	
	45	52.6, CH	4.23, m	44-NH, 46, 47, 48, 49	
	46	40.8, CH_2_	1.85, 1.47, m	44-NH, 45, 47, 48, 49	
	47	24.8, CH	1.68, m	44-NH, 45, 46, 48, 49	
	48	23.7, CH_3_	0.92, d (6.7)	44-NH,45, 46, 47, 49	49, 47, 46
	49	21.9, CH_3_	0.85, d (6.6)	44-NH, 45, 46, 47, 48	48, 47, 46
	50	ND, C			

^a^ Carbon shifts inferred from HSQC experiment. ^b^ Select carbon inferred from HMBC correlations. Missing carbon shifts marked as not detected (ND).

**Table 2:** Major MS2 fragments of acyl-surugamide A2, their predicted fragment sequence, the equivalent fragment mass for surugamide A [1,21], and the mass difference of the fragments.

MS2 Fragments of Acyl-Surugamide A2, *m*/*z*	Fragment Amino Acid Sequence	Equivalent MS2 Fragments Surugamide A, *m*/*z*	Mass Difference, *m*/*z*
841	KIFLIAI-	799	42
728	KIFLIA--	686	42
657	KIFLI---	615	42
544	KIFL----	502	42
431	KIF-----	ND	
284	KI------	ND	
397	K-----II	373	42
671	--FLIAII	671	0
581	KI---AII	539	42
558	--FLIAI-	558	0
468	K----AII	426	42
374	--FLI---	374	0
298	-----AII	298	0
261	--FL----	261	0
185	-----IA-	185	0

**Table 3:** The annotated *S. albidoflavus sur* genes and their function category predicted (Pfam) functions based on identifiable sequences from BlastN NCBI [36], and the literature annotation of the *sur* homologs. Gene functiond grouped by color; transport genes blue, core biosynthesis red, additional biosynthetic genes orange, regulatory green, and other gray.

	Function	Predicted Function	*sur* Homolog
1	transport	ABC transporter permease	
2	transport	ABC transporter permease	
3	other	ABC transporter substrate-binding protein	
4	other	Secreted protein	
5	transport	MFS transporter	
6	other	hypothetical protein	
7	regulatory	TetR/AcrR family transcriptional regulator	
8	transport	MFS transporter	
9	biosynthetic	non-ribosomal peptide synthase	*surA*
10	biosynthetic	non-ribosomal peptide synthase	*surB*
11	biosynthetic	non-ribosomal peptide synthase	*surC*
12	biosynthetic	non-ribosomal peptide synthase	*surD*
13	biosynthetic-additional	serine hydrolase domain-containing protein	*surE*
14	other	membrane protein	
15	regulatory	GntR family transcriptional regulator	*surR*
16	other	hypothetical protein	
17	transport	ATP-binding cassette domain-containing protein	
18	other	ABC transporter permease	
19	biosynthetic-additional	MbtH family protein	
20	biosynthetic-additional	alpha/beta hydrolase	
21	biosynthetic-additional	aldehyde dehydrogenase family protein	

## Data Availability

The genome sequences for *S. albidoflavus* RKJM-0023 are available in NCBI (genome CP133227 and plasmid CP133228). The NMR data for RKJM-0023 was deposited into MP-MRD (Xwaiting ID), while the molecular network generated by GNPS can be accessed following the URL (https://gnps.ucsd.edu/ProteoSAFe/result.jsp?view=network_displayer&componentindex=9&task=28b48abe40944e2e995f8d966d5708e4&show=true, accessed on 1 April 2021).

## Supplementary Materials

The following supporting information can be downloaded at: https://www.mdpi.com/article/10.3390/molecules29071482/s1, Figure S1: UHPLC-HR-ESIMS chromatogram for purified acyl-surugamide A2, including the UV plot, CAD detector, total ion chromatogram (TIC), extracted ion chromatogram (EIC) for acyl-surugamide A2 (*m/z* 954.6385 [M + H]^+^), and MS1 spectrum at 3.51 min; Figure S2: Proton (^1^H 600 MHz, ^13^C 150 MHz, DMSO-d6) for Acyl-Surugamide A2 (**1**); Figure S3: TOCSY (^1^H 600 MHz, ^13^C 150 MHz, DMSO-d6) for Acyl-Surugamide A2 (**1**); Figure S4: HSQC (^1^H 600 MHz, ^13^C 150 MHz, DMSO-d6) for Acyl-Surugamide A2 (**1**); Figure S5: HMBC (^1^H 600 MHz, ^13^C 150 MHz, DMSO-d6) for Acyl-Surugamide A2 (**1**); Figure S6: MS2 mirror plot comparing the MS2 spectrum of surugamide A (912) to acyl-surugamide A2 (954); Figure S7: MS2 fragment structures of key acyl-surugamide A2 fragments. The fragments are structurally grouped; in blue are the iterative fragment structures N-terminus acetyl-lysine, in purple are the identified fragments ending with C-terminus acetyl-lysine, and red are fragments with exact matches for surugamide A fragments used to confirm the sequence without acetyl-lysine; Figure S8: MUSCLE protein alignment of the epimerization domains for the sur BGCs from *S. albidoflavus* RKJM0023 (CP133227), J1074 (BGC0001792, CP004370.1) and JAMM993 (surA AXN72677.1, surD AXN72680.1), compared to the first two epimerization domains of the gramicidin BGC (BGC000367, AP008955.1). Blue annotations indicate the highly conserved active site motifs for a functional epimerization domain.
