# Supplementary material for: Discovery of Acyl-Surugamide A2 from Marine Streptomyces albidoflavus RKJM-0023—A New Cyclic Nonribosomal Peptide Containing an N-ε-acetyl-L-lysine Residue
Source: Molecules. 2024 Mar 27;29(7):1482. doi: 10.3390/molecules29071482 (PMC11012974; doi:10.3390/molecules29071482)
Supplement: Supplementary file 1 [file molecules-29-01482-s001.zip › molecules-2905847-supplementary.pdf]

Discovery of Acyl-Surugamide A2 from Marine *Streptomyces albidoflavus* RKJM-0023—A New Cyclic Nonribosomal Peptide Containing an N-ε-acetyl-L-lysine Residue

Zacharie A. Maw [1], Bradley Haltli [1,2], Jason J. Guo [3], Donna M. Baldisseri [4], Christopher Cartmell [5,*] and Russell G. Kerr [1,2,6,*]

1 Department of Biomedical Sciences, Atlantic Veterinary College, University of Prince Edward Island, Charlottetown, PE C1A 4P3, Canada; zmaw@upei.ca (Z.A.M.)
2 Nautilus Biosciences, Croda Canada Limited, Charlottetown, PE C1A 4P3, Canada
3 Department of Chemistry & Chemical Biology, Barnett Institute for Chemical and Biological Analysis, Northeastern University, Boston, MA 02115, USA
4 Bruker Biospin Corp., 15 Fortune Drive, Billerica, MA 01821, USA
5 Department of Pharmacology, Comprehensive Center for Pain & Addiction, College of Medicine, University of Arizona, Tucson, AZ 85724, USA
6 Department of Chemistry, University of Prince Edward Island, Charlottetown, PE C1A 4P3, Canada
* Correspondence: cartmell@arizona.edu (C.C.); rkerr@upei.ca (R.G.K.)

Supporting Information

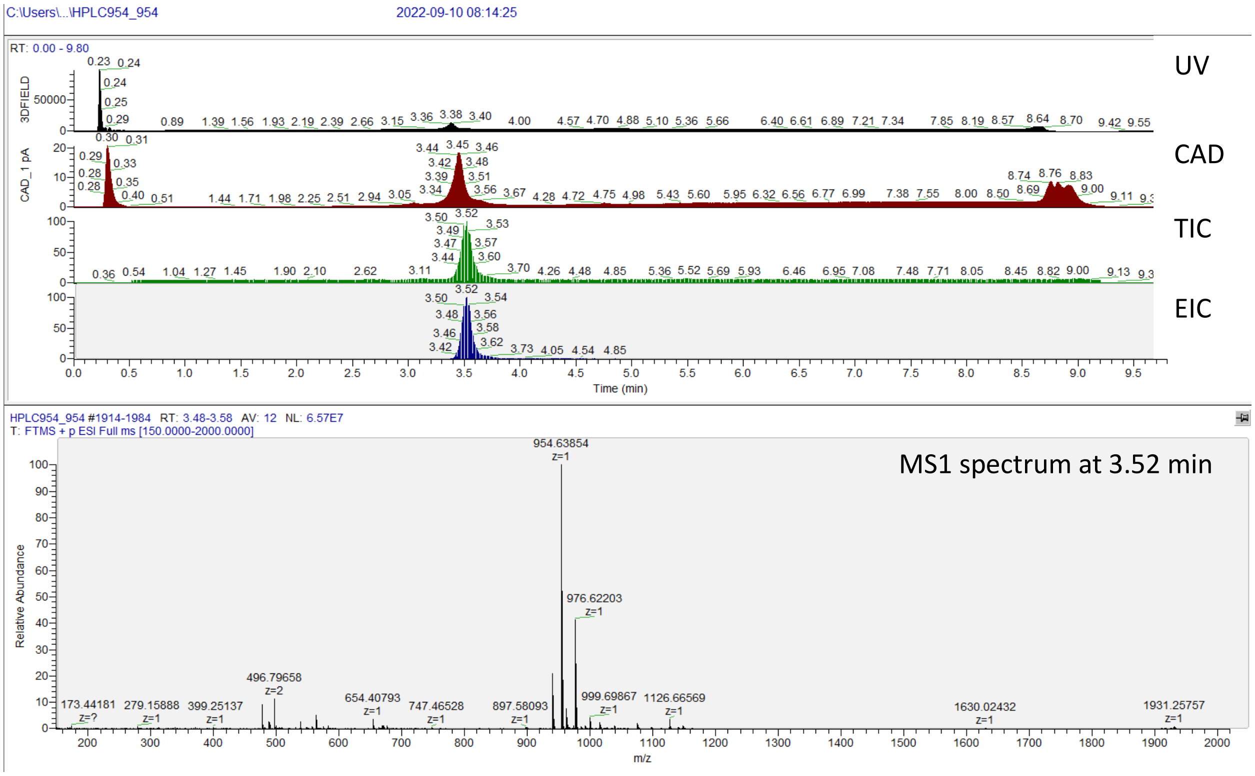

Figure S1. UHPLC-HR-ESIMS chromatogram for purified acyl-surugamide A2, including the UV plot, CAD detector, total ion chromatogram (TIC), extracted ion chromatogram (EIC) for acyl-surugamide A2 (*m/z* 954.6385 $[M+H]^{+}$), and MS1 spectrum at 3.51 minutes.

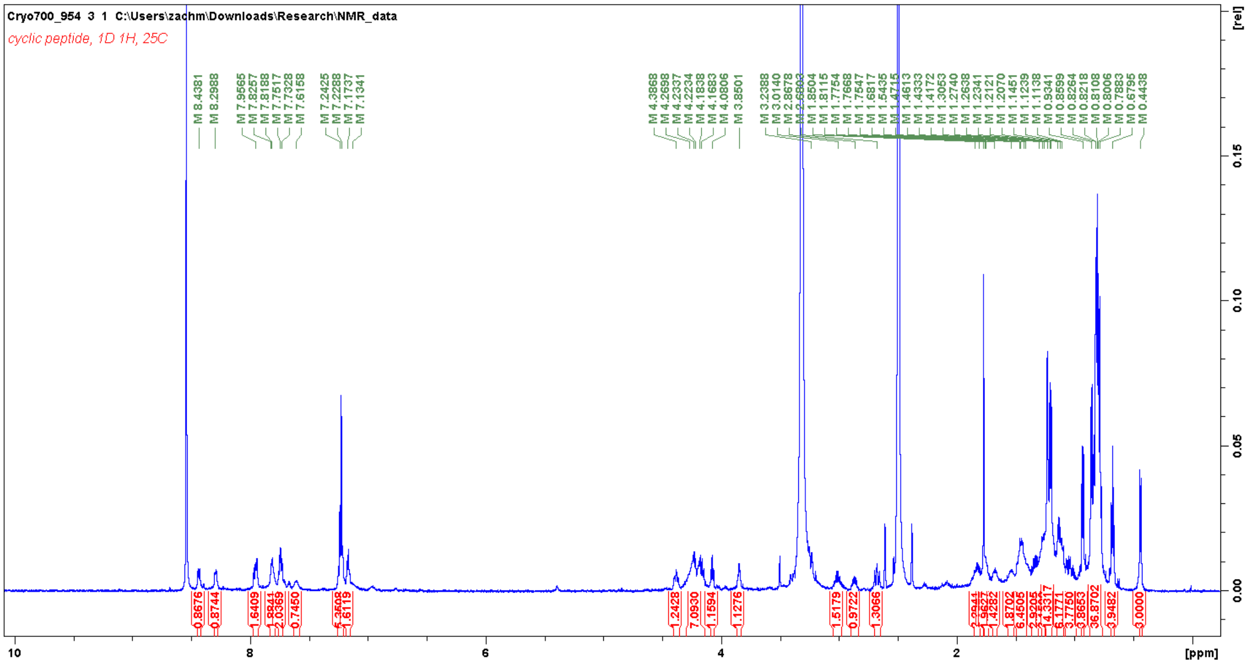

Figure S2. Proton (^{1}H 600 MHz, ^{13}C 150 MHz, DMSO-*d*$_6$) for Acyl-Surugamide A2 (**1**).

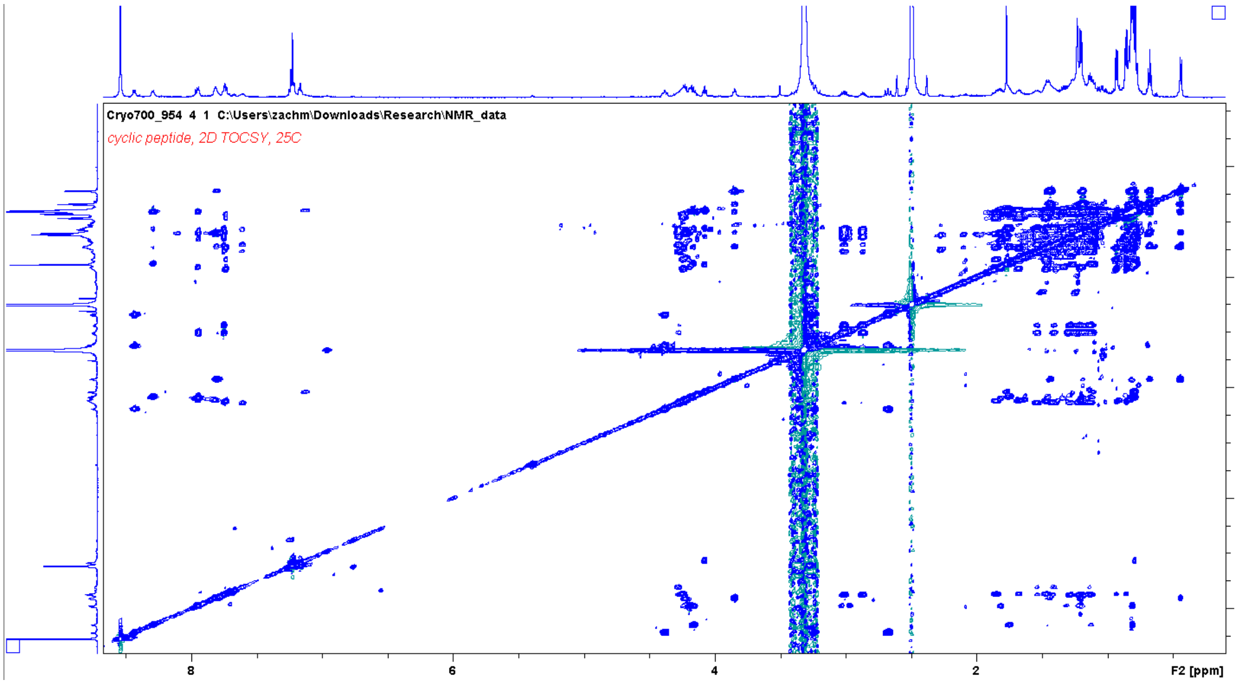

Figure S3. TOCSY (^{1}H 600 MHz, ^{13}C 150 MHz, DMSO-*d*$_6$) for Acyl-Surugamide A2 (**1**).

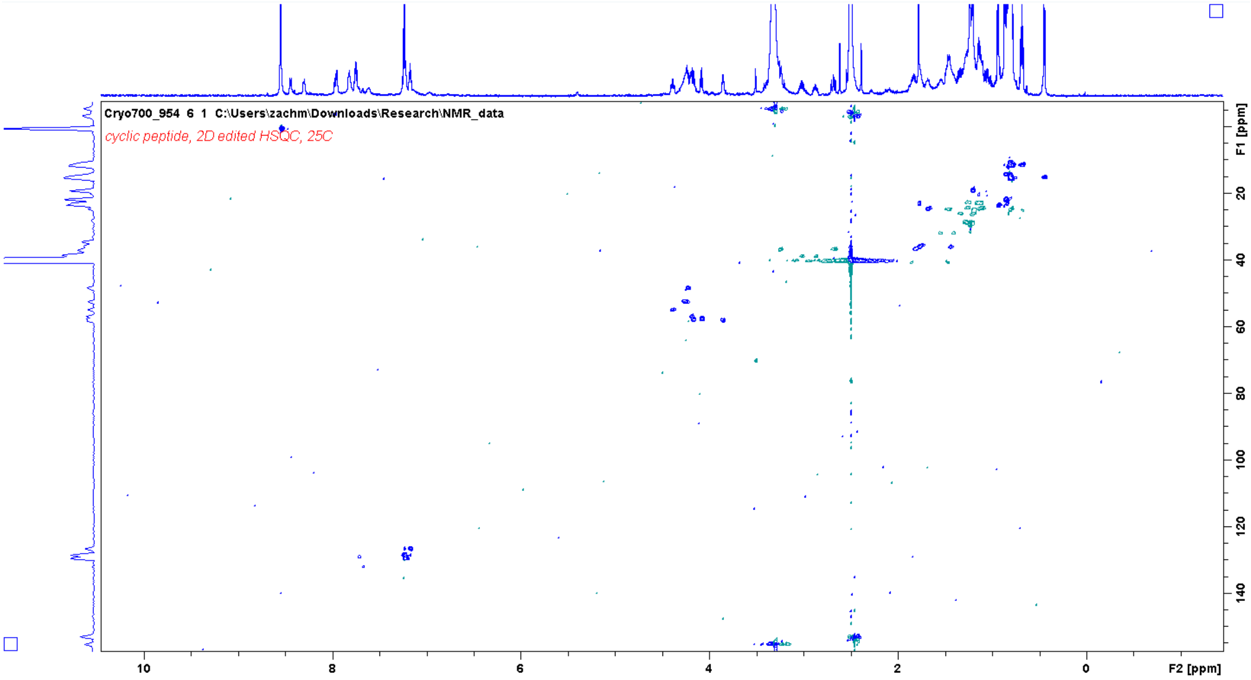

Figure S4. HSQC (^{1}H 600 MHz, ^{13}C 150 MHz, DMSO-d_6) for Acyl-Surugamide A2 (**1**).

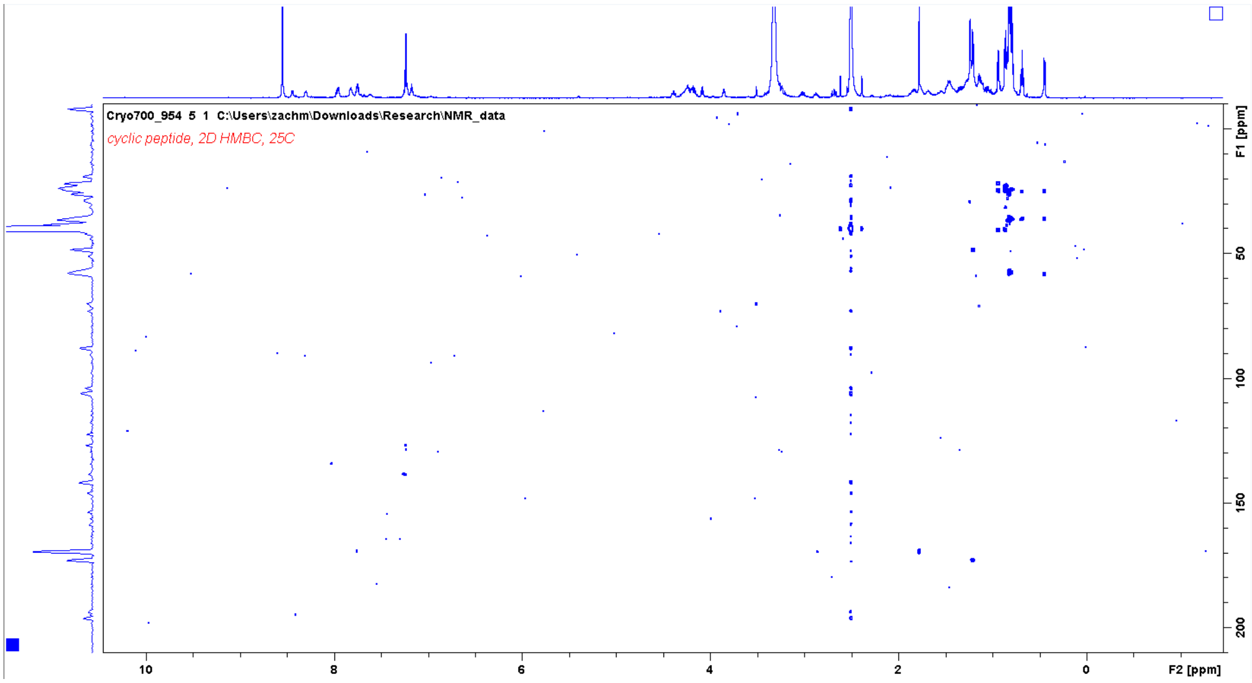

Figure S5. HMBC (^{1}H 600 MHz, ^{13}C 150 MHz, DMSO-d_6) for Acyl-Surugamide A2 (**1**).

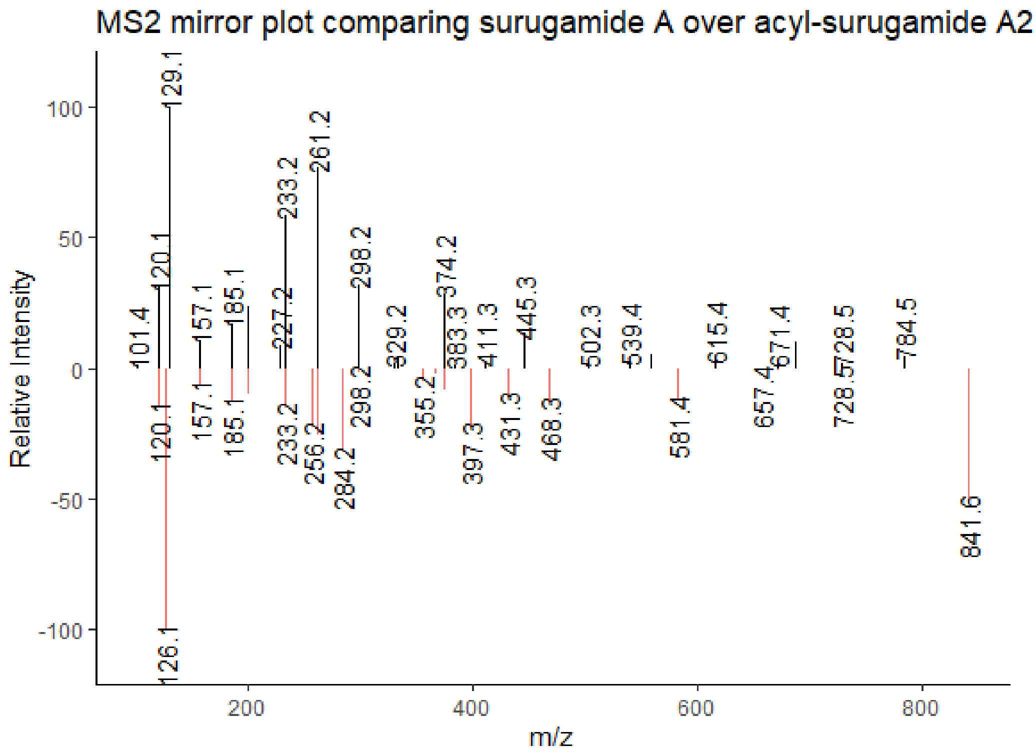

Figure S6. MS2 mirror plot comparing the MS2 spectrum of surugamide A (912) to acyl-surugamide A2 (954).

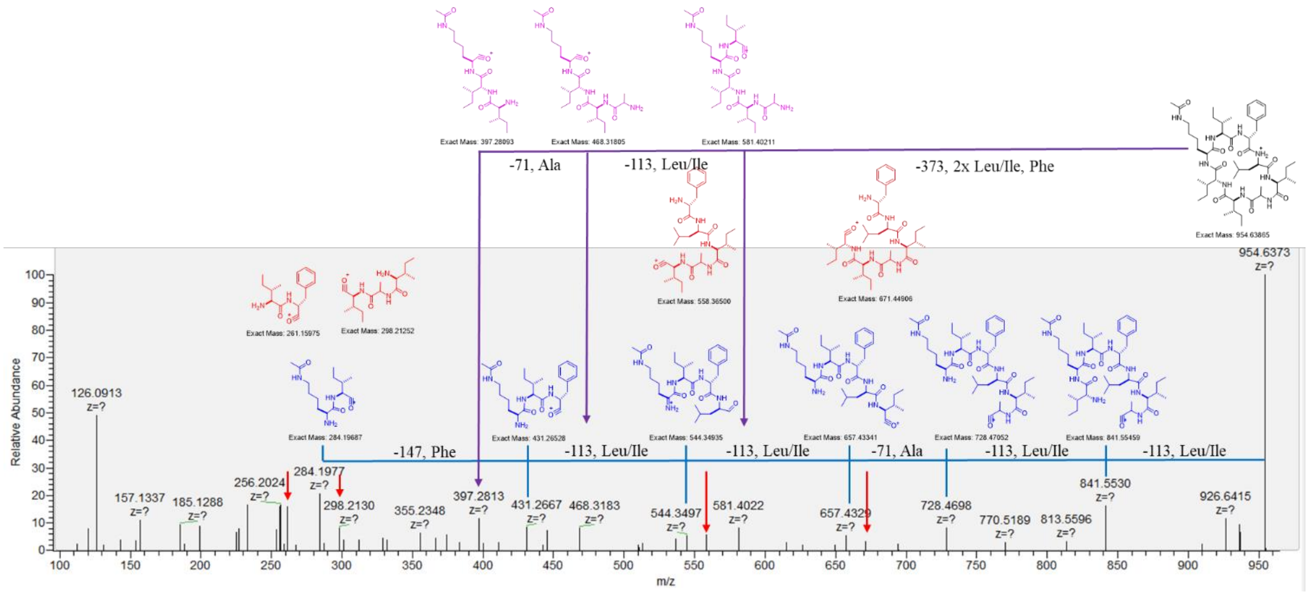

Figure S7. MS2 fragment structures of key acyl-surugamide A2 fragments. The fragments are structurally grouped; in blue are the iterative fragment structures N-terminus acetyl-lysine, in purple are the identified fragments ending with C-terminus acetyl-lysine, and red are fragments with exact matches for surugamide A fragments used to confirm the sequence without acetyl-lysine.

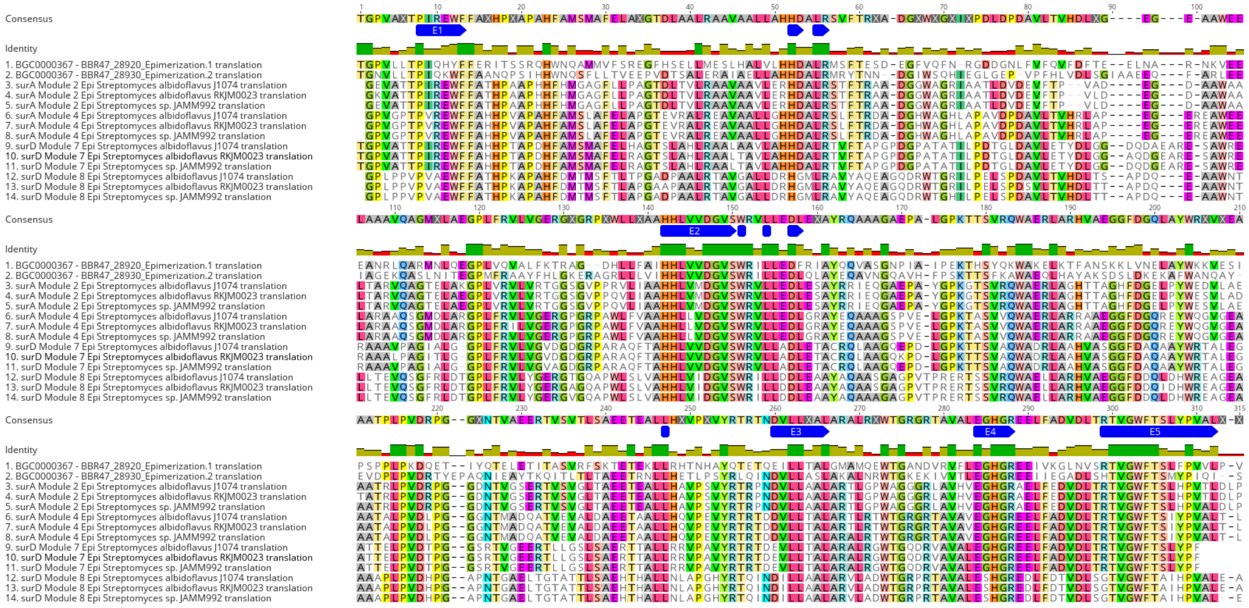

Figure S8. MUSCLE protein alignment of the epimerization domains for the *sur* BGCs from *S. albidoflavus* RKJM0023 (CP133227), J1074 (BGC0001792, CP004370.1) and JAMM993 (*sur*A AXN72677.1, *sur*D AXN72680.1), compared to the first two epimerization domains of the gramicidin BGC (BGC000367, AP008955.1). Blue annotations indicate the highly conserved active site motifs for a functional epimerization domain.
